# Survival benefit of inhaled corticosteroids in patients with chronic obstructive pulmonary disease: a nationwide cohort study

**DOI:** 10.1038/s41598-024-65763-1

**Published:** 2024-06-26

**Authors:** Jiyoung Shin, Sojung Park, Ji-Young Lee, Jin Hwa Lee

**Affiliations:** 1 Department of Health Care Policy Research, Korea Institute for Health and Social Affairs, Sejong, Republic of Korea; 2 Division of Pulmonary and Critical Care Medicine, Department of Internal Medicine, College of Medicine, Ewha Womans University, 25 Magokdong-ro 2-gil, Gangseo-gu, Seoul, 07804 Republic of Korea; 3 Inflammation-Cancer Microenvironment Research Center, College of Medicine, Ewha Womans University, Seoul, Republic of Korea

**Keywords:** Bronchodilator, Chronic obstructive pulmonary disease, Corticosteroid, Mortality, Survival, Outcomes research, Respiratory tract diseases

## Abstract

The role of inhaled corticosteroids (ICS) in chronic obstructive pulmonary disease (COPD) is debated. We investigated whether the administration of ICS could lower the mortality risk in patients with COPD. We utilized the Korean National Health Insurance Service-National Sample Cohort database from 2002 to 2019. We included patients who had claim codes for COPD and inhalation respiratory medicine at least twice a year. A time-dependent Cox regression model was employed to estimate the association between ICS usage and survival. The cumulative dose of ICS was classified into three groups, and the mortality risk was compared among these groups. Of 16,463 included patients, there were 4395 (26.7%) deaths during the mean follow-up period of 5.0 years. The time-dependent Cox regression model demonstrated that ICS users had a significantly lower mortality risk compared to non-users (adjusted hazard ratio, 0.89; 95% CI, 0.83–0.94; p < 0.001), particularly among individuals aged ≥ 55 years, women, never smokers, and those with history of asthma or coronary heart disease. Higher cumulative dose groups were associated with a lower mortality risk compared to the lowest cumulative dose group. In conclusion, the administration of ICS seemed to be associated with a lower mortality risk in patients with COPD.

## Introduction

Chronic obstructive pulmonary disease (COPD) is a leading cause of death worldwide^1,2^. The pharmacological treatment of COPD aims to relieve symptoms, improve quality of life, and prevent acute exacerbations (AE). Bronchodilators, such as long-acting muscarinic antagonists (LAMAs) and long-acting beta2-agonists (LABAs), are the mainstay of COPD treatment^1^.

Inhaled corticosteroids (ICS) are the most commonly used anti-inflammatory drugs for the treatment of COPD. Although the use of ICS is known to increase the risk of respiratory infections, it has the benefit of reducing AE rate and improve quality of life^3,4^. Several recent randomized controlled trials (RCTs) and a meta-analysis have reported that inhaled therapy containing ICS reduces all-cause mortality in patients with COPD^3–8^. Therefore, the Global Initiative for Chronic Obstructive Lung Disease guideline recommends adding ICS to LAMA and/or LABA in COPD patients with a history of frequent and/or severe exacerbations, high blood eosinophil count, and history of, or concomitant asthma^1^. However, the rates of all-cause mortality varied widely across previous RCTs comparing inhaled therapies with and without ICS^3,9^. Different eligibility criteria and treatment regimens across studies might have caused variations in all-cause mortality results. Most importantly, due to the strict eligibility criteria of RCTs, treatment outcomes or all-cause mortality rates may differ from those in real-world clinical settings.

Therefore, we aimed to investigate the survival benefits of ICS in COPD patients in a real-world clinical setting using data from the Korean National Health Insurance Service-National Sample Cohort (NHIS-NSC) database version 2.2. In addition, we investigated the potential subgroups of patients who might benefit.

## Methods

### Data source

The Korea NHIS constructed the National Health Information Database based on the claim data from 2000. This database includes demographic and other medical claims data for almost the entire Korean population (> 97%). In 2015, the NHIS constructed the NHIS-NSC population-based data, which has a follow-up period from 2002 to 2013^10^. In 2021, NHIS released NHIS-NSC version 2.2, which covers a follow-up period from 2002 to 2019^11^. In this cohort, about one million subjects were extracted using a systematic, stratified, random sampling method with 2142 strata, constructed based on age groups, sex, residential area, eligibility status and income level, from the target population of 48,222,537 individuals^12^.

### Study population

We included patients who had claim codes for COPD at least twice a year, based on the Korean Classification of Diseases, 6th revision (KCD-6), a modified version of the International Classification of Disease, 10th revision. The KCD-6 code criteria for COPD include J42.x to J44.x except J430 (eTable 1 in supplement 1). We only included patients who were prescribed inhalation respiratory medicine at least twice a year. The inhaled respiratory medicines included ICS (beclomethasone, budesonide, ciclesonide, flunisolide, fluticasone, or triamcinolone); LAMA (tiotropium, aclidinium, glycopyrronium, umeclidinium); LABA (formoterol or salmeterol); a combination of ICS/LABA; a combination of LAMA/LABA; short-acting beta2-agonists (fenoterol, procaterol, salbutamol, or terbutaline); short-acting muscarinic antagonists (ipratropium); and a combination of short-acting beta2- agonists and short-acting muscarinic antagonists. Patients with claim codes for COPD between 2002 and 2003 were excluded to allow for a washout period, as they may have been diagnosed with COPD before 2002. The index date, the beginning of the follow-up period, was defined as the date the inhaler was first prescribed. The time before the use of ICS was allocated to non-ICS users to address the immortal time bias. Since the date of death contained only the year and month but no day, it was assumed to be the 15th of the month of death. The cutoff date for data was December 31, 2019.

**Figure 1:**
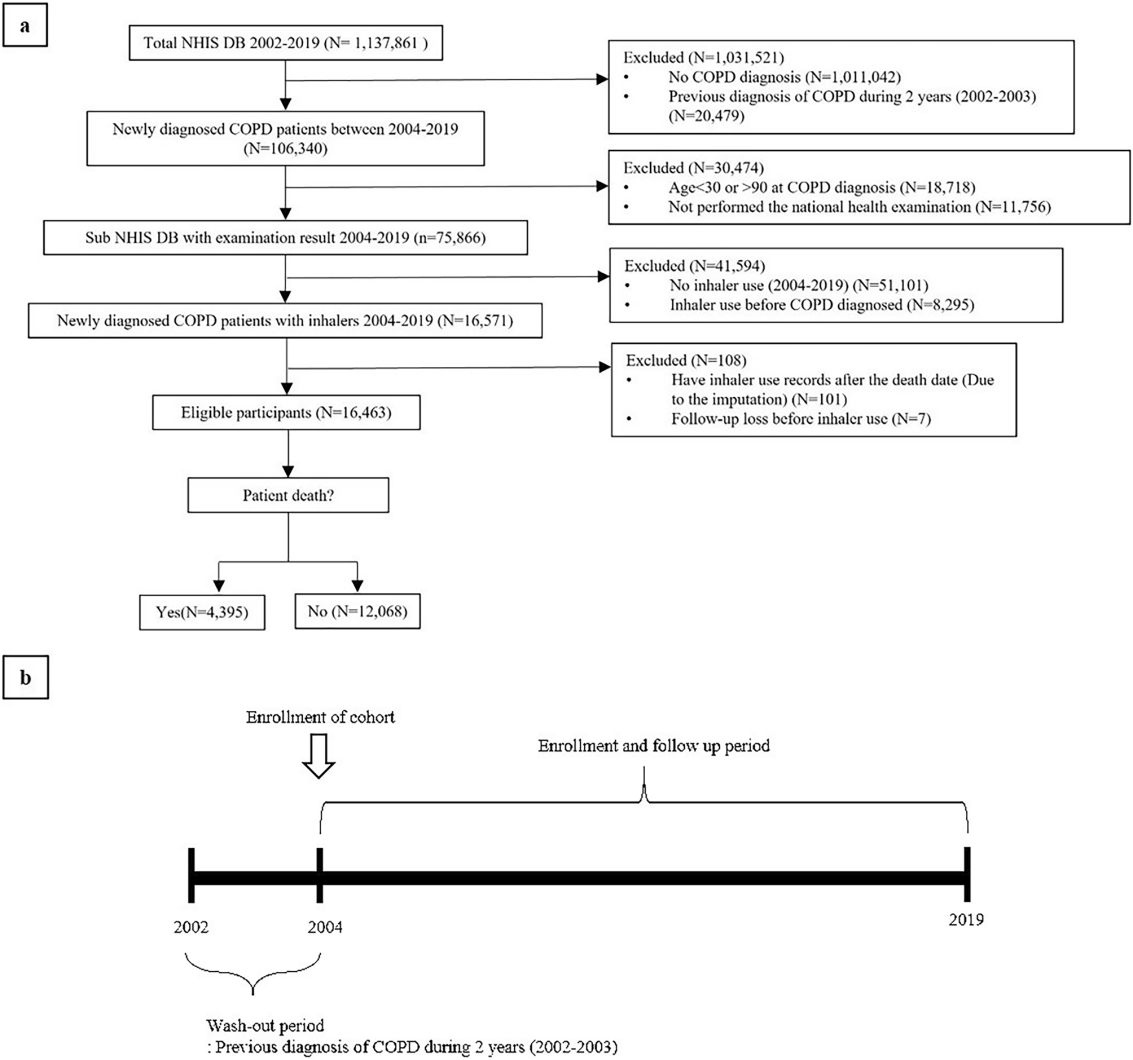
Study design. Population selection diagram of the study (**a**) and study design over time (**b**). *NHIS DB* National Health Insurance Service database, *COPD* chronic obstructive lung disease.

Patients younger than 30 years old or older than 90 years old, those without a national health examination record, and those who used inhaler before being diagnosed with COPD were excluded. Since the date of death was recorded as the 15th of every month, 101 patients had claim codes for inhaler prescriptions after estimated date of death. These patients were also excluded from the study. Finally, out of 16,571 patients with COPD, 16,463 were included in the present study (Fig. 1).

### Study design

The following variables were assessed: demographic data, general health examinations, and medical treatments. General health examinations, including lifestyle and health behavior, were obtained through questionnaires from nationwide health examinations conducted by the NHIS. The cumulative dose of ICS was calculated by aggregating the total dose of ICS medication prescribed during each patient’s follow-up and then applying a natural log transformed. The equivalent doses of ICS were 100 mg beclomethasone, 50 mg beclomethasone hydrofluoralkane, 80 mg budesonide, 200 mg triamcinolone, 32 mg ciclesonide, 50 mg fluticasone and 200 mg flunisolide. We compared the mortality risk depending on ICS usage. Moreover, the cumulative dose of ICS was classified into three groups: low (< 3937.5 μg), intermediate (3937.5 μg < and < 48,437.5 μg) and high (> 48,437.5 μg), based on the 33.3 percentile cut-off point, and the mortality risk was compared among these groups. For the covariates, we used demographic characteristics including age, sex, body mass index (BMI) and household income level. We also included smoking status and Charlson comorbidity index (CCI) as covariates. All covariates were considered as mortality risk factors, and these variables were assessed from the latest record of the national health examination from the year of COPD diagnosis. We performed subgroup analyses based on the patients’ age, sex, and smoking status. Additionally, subgroup analyses based on the comorbidity status of asthma and coronary heart disease (CHD) diagnosed before inhaler initiation were performed.

All methods were performed in accordance with the relevant guidelines and were approved by the Institutional Review Board of Ewha Womans University Hospital, Seoul, Republic of Korea (IRB number: SEUMC 2023-03-002). Informed consent from participants was waived by IRB because NHIS-NSC does not contain any identifying information, and the study involved minimal risk to human subjects.

### Statistical analysis

Categorical characteristics between survivors and non-survivors were compared by using the χ^2^ test, and continuous characteristics were compared by using the *t*-test. The Kaplan–Meier curves were established based on the use of ICS, sex, cumulative dose of ICS, and smoking status, with significance determined by the log-rank test. A time-dependent Cox regression model was used to estimate the association between ICS usage and death in COPD patients, with the adjusted hazard ratio (aHR) and 95% confidence interval (CI) being calculated. For non-ICS users and ICS users prior to the first ICS prescription, the cumulative dose was considered zero. All analyses were conducted using SAS version 9.4 software (SAS Institute, Cary, NC, USA) and R Studio 1.0.136 (R Studio, Inc). All p-values were two-sided, and those < 0.05 were considered statistically significant.

## Results

### Baseline characteristics

The present study included 16,463 patients, with a mean age of 63.2 years. Of these, 55.9% were men, as detailed in Table 1. During the mean follow-up of 5.0 years from the date of first diagnosis of COPD to the date of death or censoring, there were 4395 (26.7%) deaths. The proportion of patients over 55 years of age was significantly higher in the non-survivor group (95.1%) compared with the survivor group (69.1%, p < 0.001). Survivors had significantly low CCI (2.1 ± 1.4 vs. 3.1 ± 2.0, p < 0.001) and used more ICS or LABA-containing inhaled therapy than non-survivors. A total of 35.4% of patients with COPD had been diagnosed with CHD, showing a significant difference in the prevalence of CHD between the two groups (42.0% vs. 33.0%, p < 0.001).

**Table 1:** Baseline characteristics of the study subjects.

	Total	Survivor	Non-survivor	P
	n = 16,463	n = 12,068	n = 4395	
Follow up period, years	5.0 ± 4.2	5.7 ± 4.2	3.0 ± 3.3	< 0.001
Age, years	63.2 ± 12.8	60.3 ± 12.8	71.0 ± 9.0	< 0.001
≥ 55	12,521 (76.1)	8343 (69.1)	4178 (95.1)	< 0.001
Sex, men	9209 (55.9)	6109 (50.6)	3100 (70.5)	< 0.001
Smoking status				< 0.001
Never smokers	9669 (58.7)	7355 (61.0)	2314 (52.7)	
Former smokers	2713 (16.5)	1963 (16.3)	750 (17.1)	
Current smokers	4081 (24.8)	2750 (22.8)	1331 (30.3)	
BMI, kg/m^2^	23.7 ± 3.6	24.1 ± 3.6	22.8 ± 3.6	< 0.001
CCI	2.4 ± 1.6	2.1 ± 1.4	3.1 ± 2.0	< 0.001
Household income levels				< 0.001
0–20%	2542 (15.4)	1904 (15.8)	638 (14.5)	
20–40%	2115 (12.9)	1577 (13.1)	538 (12.2)	
40–60%	2697 (16.4)	2018 (16.7)	679 (15.5)	
60–80%	3489 (21.2)	2577 (21.4)	912 (20.8)	
80–100%	4588 (27.9)	3236 (26.8)	1352 (30.8)	
Respiratory medication				
ICS	7292 (44.3)	5471 (45.3)	1821 (41.4)	< 0.001
ICS/LABA	6394 (38.8)	5077 (42.1)	1317 (30.0)	< 0.001
LAMA	3311 (20.1)	2201 (18.2)	1110 (25.3)	< 0.001
LAMA/LABA	1829 (11.1)	1565 (13.0)	264 (6.0)	< 0.001
LABA	1502 (9.1)	1244 (10.3)	258 (5.9)	< 0.001
SABA	12,451 (75.6)	8734 (72.4)	3717 (84.6)	< 0.001
SAMA	7098 (43.1)	3992 (33.1)	3106 (70.7)	< 0.001
SABA/SAMA	148 (0.9)	68 (0.6)	80 (1.8)	< 0.001
Underlying disease				
Asthma	12,678 (77.0)	9617 (79.7)	3061 (69.7)	< 0.001
CHD	5829 (35.4)	3983 (33.0)	1846 (42.0)	< 0.001

Data are shown as means ± standard deviations or n (%) per each group.

*BMI* body mass index, *CCI* Charlson comorbidity index, *ICS* inhaled corticosteroids, *LABA* long-acting beta-2 agonists, *LAMA* long-acting muscarinic antagonists, *SABA* short-acting beta-2 agonists, *SAMA* short-acting muscarinic antagonists, *CHD* coronary heart disease.

### ICS usage and mortality

**Figure 2:**
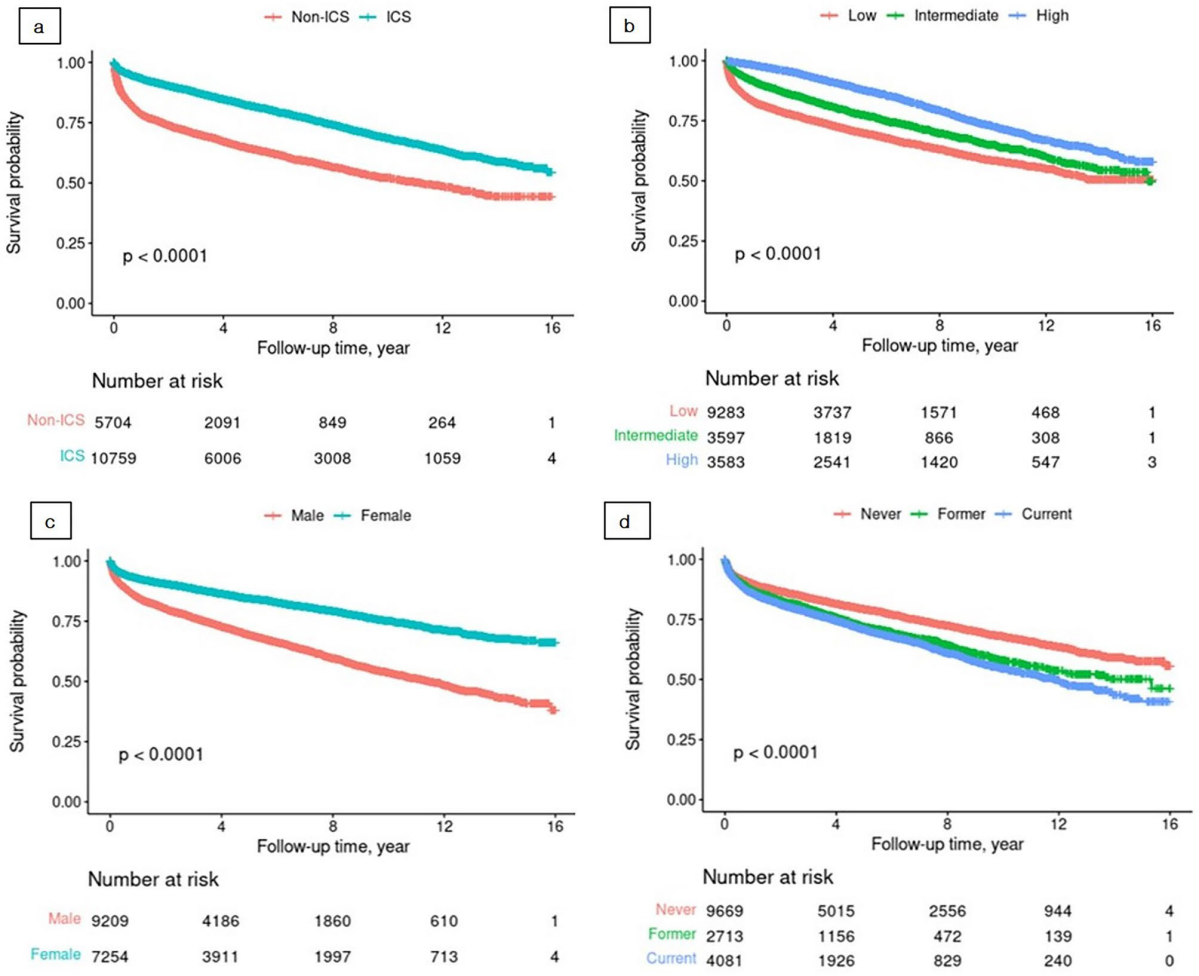
Kaplan–Meier curves of study population. Survival according to use of inhaled corticosteroids (**a**), cumulative dose of inhaled corticosteroids (**b**), sex (**c**), and smoking status (**d**) among patients with chronic obstructive pulmonary disease.

A total of 10,759 patients (65.4%) used inhaled therapy containing ICS. The Kaplan–Meier curves indicated that the use of ICS and the cumulative dose of ICS did not violate the proportional assumption. As a result, the Kaplan–Meier curves showed that ICS users (Fig. 2a) and the intermediate and high dose ICS groups (Fig. 2b) had significantly improved survival compared with non-ICS users and low dose ICS group, respectively. Moreover, men (Fig. 2c) and current smokers (Fig. 2d) had a significantly lower survival probability compared with women and former or never smokers, respectively.

The crude models of time-dependent Cox regression model showed that the use of ICS was associated with a lower risk of mortality in the overall population (HR, 0.69; 95% CI, 0.65–0.73, p < 0.001, Table 2). The adjusted models performed by adjusting for age, sex, BMI, household income level, CCI, and smoking status revealed that the use of ICS was associated with a lower risk of mortality in the overall population (aHR, 0.89; 95% CI, 0.83–0.94, p < 0.001). Especially, patients aged ≥ 55 years, women, never smokers, and those with history of asthma or CHD showed a lower risk of mortality.

**Table 2:** Result of time-dependent Cox regression between use of inhaled corticosteroids and mortality risk in patients with chronic obstructive pulmonary disease.

	Crude hazard ratio	P	Adjusted hazard ratio	P
	(95% CI)		(95% CI)	
Total^a^	0.69 (0.65–0.73)	< 0.001	0.89 (0.83–0.94)	< 0.001
Age ≥ 55 years^a^	0.77 (0.73–0.82)	< 0.001	0.90 (0.84–0.96)	< 0.001
Women^b^	0.53 (0.47–0.59)	< 0.001	0.70 (0.62–0.78)	< 0.001
Never smoker^c^	0.60 (0.55–0.65)	< 0.001	0.81 (0.75–0.88)	< 0.001
Former smoker^c^	0.82 (0.71–0.95)	0.01	0.95 (0.82–1.11)	0.5229
Current smoker^c^	0.82 (0.73–0.91)	< 0.001	1.01 (0.91–1.13)	0.837
Asthma^a^	0.70 (0.65–0.75)	< 0.001	0.87 (0.81–0.94)	< 0.001
Cardiovascular disease^a^	0.69 (0.63–0.76)	< 0.001	0.82 (0.75–0.90)	< 0.001

^a^Adjusted hazard ratios were adjusted for age, sex, body mass index, household income level, Charlson comorbidity index, and smoking status.

^b^Adjusted hazard ratios were adjusted for age, body mass index, household income level, Charlson comorbidity index, and smoking status.

^c^Adjusted hazard ratios were adjusted for age, sex, body mass index, household income level and Charlson comorbidity index.

**Figure 3:**
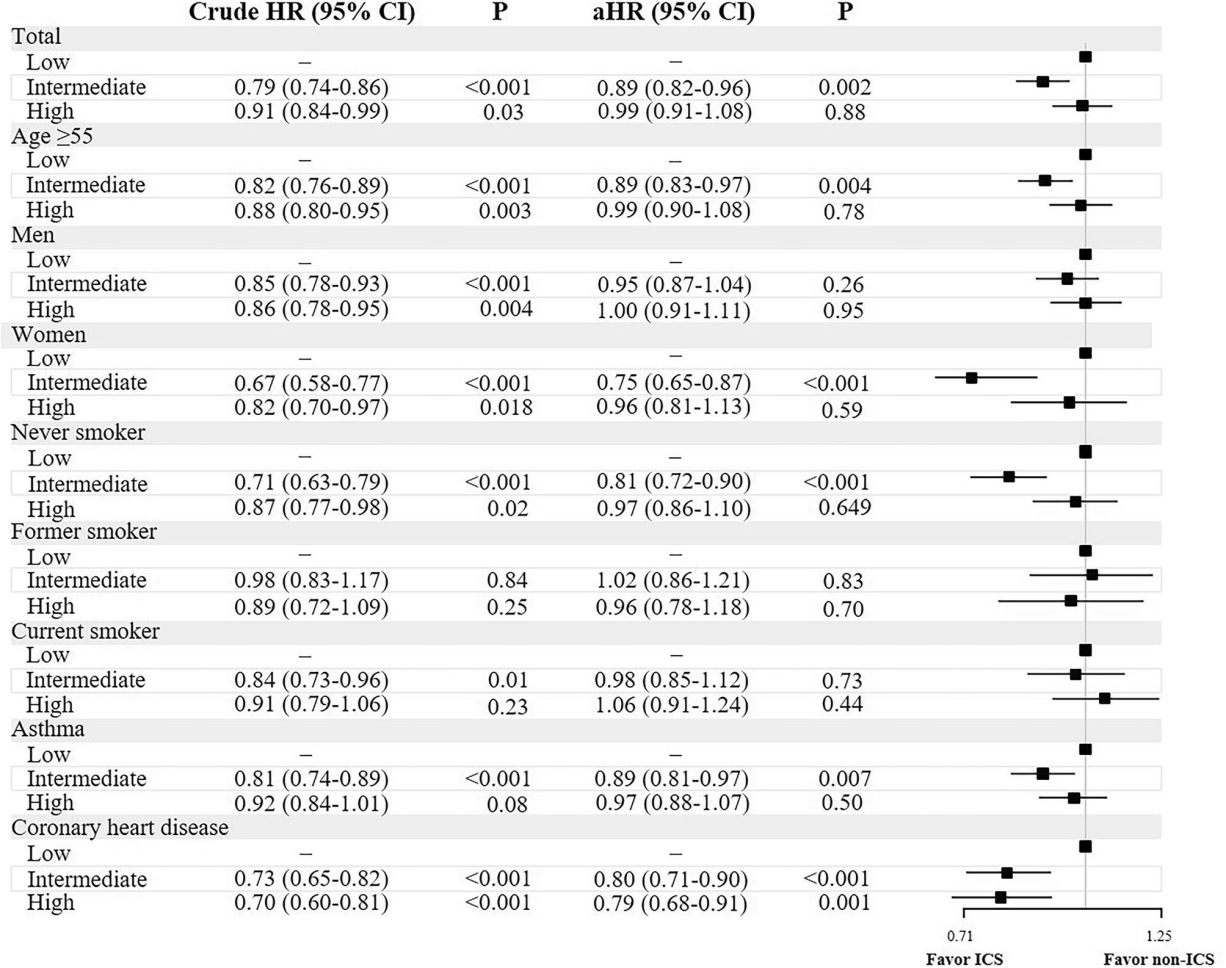
Association among three cumulative dose of inhaled corticosteroids exposure groups and the mortality risk. Models were adjusted for age, sex, body mass index, household income level, Charlson comorbidity index, and smoking status. Inhaled corticosteroids usage was converted to μg, and analyzed as a time-dependent covariate.

In the analysis with three groups of the cumulative dose of ICS, the intermediate (HR, 0.79; 95% CI, 0.74–0.86, p < 0.001) and high dose groups (HR, 0.91; 95% CI, 0.84–0.99, p = 0.03, Fig. 3) showed significantly lower mortality risk in the overall population. In subgroup analysis using the adjusted models, the intermediate dose group showed significantly lower mortality risk in patients aged ≥ 55 years, women, never smokers, and those with history of asthma or CHD, compared with the low dose group. The adjusted models of time-dependent Cox regression showed that a cumulative dose of ICS was associated with a lower risk of mortality in the overall population (HR, 0.991; 95% CI, 0.985–0.997, p = 0.004, eTable 2 in supplement 1).

## Discussion

The present study demonstrated that inhaled therapy containing ICS was associated with a reduction in all-cause mortality risk in patients with COPD. To the best of our knowledge, this is the first study to show the benefits of ICS in improving survival in COPD, using nationwide cohort data from real-world practice. In subgroup analysis, an intermediate cumulative dose of ICS showed the most significant improvement in survival and a pronounced reduction in mortality risk among patients aged ≥ 55 years, women, never smokers, and those with history of asthma or CHD.

The specific mechanism by which ICS reduces the risk of all-cause mortality in patients with COPD remains unclear. ICSs are anti‐inflammatory drugs that have been proven to reduce symptoms, the rate of decline in forced expiratory volume in one second and the AE rate when used with bronchodilators^4,13–18^. AE is one of the most common causes of death in patients with COPD, with in-hospital mortality rates after hospitalization for a COPD AE ranging from 2.5 to 15%^19–22^. Because inflammation plays an important role in the development of AE, the anti-inflammatory effect of ICS may reduce mortality by preventing AEs. Additionally, the anti-inflammatory effect of ICS might also favorably affect airway remodeling, as demonstrated by the decline in forced expiratory volume in one second over time^4,23^. We demonstrated that ICS was less effective in current and former smokers who might have a high inflammatory burden and a high risk of ongoing airway remodeling compared to never smokers. Even if ICSs are used, inflammation will inevitably persist if patients are exposed to continuous stimulation such as smoking. On the other hand, delaying the deterioration of lung function might reduce the pulmonary vascular burden, especially in patients with severe COPD who usually suffer from frequent AEs or comorbidities^24^.

In the present study, patients with concomitant CHD showed more benefit from ICS. The post hoc analysis of TORCH (Towards a Revolution in COPD Health) showed similar results, indicating that the ICS/LABA combination had a beneficial effect on reducing CHD events in subgroups who had previously had a myocardial infarction^25^. Several studies showed that ICS was associated with a reduction in CHD events and CHD-related mortality in patients with COPD^26–28^. It is known that the risk of CHD increases after an AE of COPD^29^. Corticosteroids not only prevent AE but also upregulate beta2-adrenoreceptors and can prevent or reverse down-regulation of beta2-adrenoreceptors in response to agonists^30–32^. Dransfield et al. reported that ICS/LABA may reduce aortic pulse wave velocity in those with elevated arterial stiffness^33^. Rabe et al. reported that a high cumulative dose of ICS was associated with a greater effect in reducing CHD risk compared with a low cumulative dose of ICS^24^. Although inhaled delivery of corticosteroids provides minimized systemic anti-inflammatory effects, ICS might also have a cardio-protective effect because cardiovascular disease is provoked by chronic inflammation^34^.

The most concern regarding the use of ICS is the increased risk of pneumonia. High-dose ICSs have been associated with a high pneumonia risk, which offsets its anti-inflammatory effect^35–37^. The meta-analysis showed that the use of ICS increases the incidence of pneumonia and serious pneumonia, but not pneumonia-related mortality^8^. Some studies have shown that the incidence of pneumonia depends on the type of ICS; budesonide was found to cause less pneumonia compared with fluticasone^36^. Chen et al. reported that medium- and low-dose ICSs, rather than high-dose ICSs, were associated with a significant reduction in all-cause mortality^8^. Consistent with this result, the present study showed that intermediate-dose ICS had the lowest mortality, although high-dose ICS showed a decrease in mortality risk.

The present study has several limitations. First, although we defined the diagnosis and outcome using a structural definition, the possibility of over-diagnosis or misdiagnosis could not be totally excluded. This is because the database does not include pulmonary function tests, respiratory symptoms, and laboratory tests, leading us to define COPD using claim data. Second, the proportions of women and never smokers were relatively high compared to the known epidemiologic characteristics of COPD patients in Korea^38^. This may be due to the diagnosis of COPD using claim codes. In addition, we excluded people who did not receive a national examination, which is generally known that smokers have lower interest in healthcare and therefore have lower rates of national examinations compared with never smokers. However, the relatively high proportions of women and never smokers can be considered a positive reflection of the characteristics of Korean COPD patients, especially since many women with COPD suffer from conditions caused by tuberculosis-destroyed lung or bronchiectasis. Furthermore, women and never smokers are more likely to have COPD-asthma overlap, which may have resulted in a more pronounced effect of ICS^39^. Third, the effect of ICS alone cannot be fully investigated because ICS was mainly used with other bronchodilators. However, since bronchodilators are the mainstay of treatment for COPD, the additive effect of ICS with LABA or LAMA/LABA is clinically significant. Fourth, smoking status and age may be important prognostic factors for death or a natural course of COPD. More patients in the non-survivors were former- or current smokers which may mean that they had poorer lung function or that COPD progressed more quickly. In a subgroup analysis based on smoking status, never smokers showed a higher survival rate. Therefore, smoking status might have influenced mortality rate. Age factor may also be associated with poor lung function or a short remaining lifespan.

In conclusion, the present study demonstrated that inhaled therapy containing ICS improved all-cause mortality, particularly in individuals aged ≥ 55 years, women, never smokers, and those with history of asthma or CHD. A cumulative dose of ICS was associated with improved survival, especially at an intermediate dose. Further well-designed studies providing all-cause mortality and dose and type of ICS on varied patient subgroups are needed to guide clinical practice better.

### Supplementary Information

Supplementary Tables.

## Data Availability

The NHIS-NSC data that support the findings of this study are available from the NHIS but restrictions apply to the availability of these data, which were used under license for the current study, and so are not publicly available. Data are, however, available upon reasonable request and with permission of NHIS.
