# Supplementary material for: Survival benefit of inhaled corticosteroids in patients with chronic obstructive pulmonary disease: a nationwide cohort study
Source: Sci Rep. 2024 Jun 26;14:14703. doi: 10.1038/s41598-024-65763-1 (PMC11208440; doi:10.1038/s41598-024-65763-1)
Supplement: Supplementary file 1 — Supplementary Tables. [file 41598_2024_65763_MOESM1_ESM.docx]

eTable 1. Korean Classification of Diseases, 6^th^ revision codes used for defining chronic obstructive pulmonary disease and coronary heart disease

| Disease | KCD-6 Code | Description |
| --- | --- | --- |
| Chronic obstructive lung disease (COPD) | J42 | Unspecified chronic bronchitis |
|  | J43 | Emphysema |
|  | J43.1 | Panlobular emphysema |
|  | J43.2 | Centrilobular emphysema |
|  | J43.8 | Other emphysema |
|  | J43.9 | Emphysema, unspecified |
|  | J44 | Other chronic obstructive pulmonary disease |
|  | J44.0 | Chronic obstructive pulmonary disease with acute lower respiratory infection |
|  | J44.1 | Chronic obstructive pulmonary disease with acute exacerbation, unspecified |
|  | J44.8 | Other specified chronic obstructive pulmonary disease |
|  | J44.9 | Chronic obstructive pulmonary disease, unspecified |
| Coronary heart disease (CHD) | I20 | Angina pectoris |
|  | I20.0 | Unstable angina |
|  | I20.1 | Angina pectoris with documented spasm |
|  | I20.8 | Other forms of angina pectoris |
|  | I20.9 | Angina pectoris, unspecified |
|  | I21 | Acute myocardial infarction |
|  | I21.0 | Acute transmural myocardial infarction of anterior wall |
|  | I21.1 | Acute transmural myocardial infarction of inferior wall |
|  | I21.2 | Acute transmural myocardial infarction of other sites |
|  | I21.3 | Acute transmural myocardial infarction of unspecified site |
|  | I21.4 | Acute subendocardial myocardial infarction |
|  | I21.9 | Acute myocardial infarction, unspecified |
|  | I22 | Subsequent myocardial infarction |
|  | I22.0 | Subsequent myocardial infarction of anterior wall |
|  | I22.1 | Subsequent myocardial infarction of inferior wall |
|  | I22.8 | Subsequent myocardial infarction of other sites |
|  | I22.9 | Subsequent myocardial infarction of unspecified site |
|  | I23 | Certain current complications following acute myocardial infarction |
|  | I23.0 | Haemopericardium as current complication following acute myocardial infarction |
|  | I23.1 | Atrial septal defect as current complication following acute myocardial infarction |
|  | I23.2 | Ventricular septal defect as current complication following acute myocardial infarction |
|  | I23.3 | Rupture of cardiac wall without haemopericardium as current complication following acutemyocardial infarction |
|  | I23.4 | Rupture of chordae tendineae as current complication following acute myocardial infarction |
|  | I23.5 | Rupture of papillary muscle as current complication following acute myocardial infarction |
|  | I23.6 | Thrombosis of atrium, auricular appendage, and ventricle as current complicationsfollowing acute myocardial infarction |
|  | I23.8 | Other current complications following acute myocardial infarction |
|  | I24 | Other acute ischaemic heart diseases |
|  | I24.0 | Coronary thrombosis not resulting in myocardial infarction |
|  | I24.1 | Dressler’s syndrome |
|  | I24.8 | Other forms of acute ischaemic heart disease |
|  | I24.9 | Acute ischaemic heart disease, unspecified |
|  | I25 | Chronic ischaemic heart disease |
|  | I25.0 | Atherosclerotic cardiovascular disease, so described |
|  | I25.1 | Atherosclerotic heart disease |
|  | I25.2 | Old myocardial infarction |
|  | I25.3 | Aneurysm of heart |
|  | I25.4 | Coronary artery aneurysm and dissection |
|  | I25.5 | Ischaemic cardiomyopathy |
|  | I25.6 | Silent myocardial ischaemia |
|  | I25.8 | Other forms of chronic ischaemic heart disease |
|  | I25.9 | Chronic ischaemic heart disease, unspecified |

eTable 2. Result of time-dependent Cox regression between cumulative dose of inhaled corticosteroids exposure and mortality risk in patients with chronic obstructive pulmonary disease

|  | Crude hazard ratio | P | Adjusted hazard ratio | P |
| --- | --- | --- | --- | --- |
|  | (95% CI) |  | (95% CI) |  |
| Total^a^ | 0.97 (0.96-0.97) | <0.001 | 0.991 (0.985-0.997) | 0.004 |
| Age ≥55 years^a^ | 0.98 (0.97-0.98) | <0.001 | 0.992 (0.985-0.998) | 0.009 |
| Women^b^ | 0.94 (0.93-0.95) | <0.001 | 0.97 (0.96-0.98) | <0.001 |
| Never smoker^c^ | 0.96 (0.95-0.97) | <0.001 | 0.98 (0.97-0.99) | <0.001 |
| Former smoker^c^ | 0.99 (0.97-1.00) | 0.06 | 1.00 (0.98-1.01) | 0.8 |
| Current smoker^c^ | 0.98 (0.97-0.99) | 0.002 | 1.00 (0.99-1.01) | 0.66 |
| Asthma^a^ | 0.97 (0.97-0.98) | <0.001 | 0.990 (0.983-0.998) | 0.009 |
| Cardiovascular disease^a^ | 0.96 (0.95-0.97) | <0.001 | 0.98 (0.97-0.99) | <0.001 |

^a^Adjusted hazard ratios were adjusted for age, sex, body mass index, household income level, Charlson comorbidity index, and smoking status

^b^Adjusted hazard ratios were adjusted for age, body mass index, household income level, Charlson comorbidity index, and smoking status

^c^Adjusted hazard ratios were adjusted for age, sex, body mass index, household income level, and Charlson comorbidity index
